# Is Adjuvant Chemotherapy Worthwhile After Radical Resection for Single Lung Metastasis From Colorectal Cancer? A Multicentric Analysis Evaluating the Risk of Recurrence

**DOI:** 10.3389/fonc.2019.00763

**Published:** 2019-08-20

**Authors:** Cristian Rapicetta, Filippo Lococo, Federico Davini, Francesco Carleo, Juha Kauppi, Teresa Severina Di Stefano, Sara Ricciardi, Marco Di Martino, Jari Räsänen, Massimiliano Paci, Franca Melfi, Giuseppe Cardillo

**Affiliations:** ^1^Thoracic Surgery Unit, Azienda USL di Reggio Emilia - IRCCS, Reggio Emilia, Italy; ^2^Unit of Minimally Invasive and Robotic Thoracic Surgery, Robotic Multispeciality Center for Surgery, University Hospital of Pisa, Pisa, Italy; ^3^Unit of Thoracic Surgery, San Camillo Forlanini Hospital, Rome, Italy; ^4^Unit of Thoracic Surgery, Helsinki University Central Hospital, Heart and Lung Center, Helsinki, Finland

**Keywords:** metastasis, colorectal cancer, survival, recurrence, CEA

## Abstract

**Background:** Adjuvant chemotherapy after resection of colorectal cancer (CRC) lung metastases may reduce recurrences and improve survival. The choice of best candidates for adjuvant chemotherapy in this setting is controversial, especially when a single lung metastases (SLM) is resected. The aim of this study is to evaluate the risk of recurrence after radical resection for single lung metastasis from CRC.

**Patients and methods:** Demographic, clinical, and pathological data were retrospectively collected for patients radically operated on for single pulmonary metastasis from CRC in 4 centers. Survival was computed by Kaplan-Meyer methods. Chi-square, log-rank test, and for multivariate analysis, Cox-regression and binary logistic regression were used when indicated.

**Results:** The sample consisted of 344 patients, mean age 65 yrs. Overall 5 yrs survival was 61.9%. Recurrence occurred in 113 pts (32.8%). At univariate analysis, age > 70 (*p* = 0.046) and tumor size > 2 cm (*p* = 0.038) were predictive of the worst survival chance, while synchronous lung metastasis (*p* = 0.039), previous resection of extrathoracic metastasis (*p* = 0.017), uptake at FDG-PET scan (*p* = 0.006) and short (<12 months) disease-free interval (DFI) prior to lung metastasectomy (*p* = 0.048) were risk factors for recurrence. At multivariate analysis, only high CEA (>4 ng/mL) was associated with worst survival (HR: 4.3, *p* = 0.014), while prior abdominal surgery (HR: 3, *p* = 0.033), PET positivity (HR: 2.7, *p* = 0.041), and DFI > 12 months (HR: 0.14, *p* < 0.001) confirmed to predict recurrence of disease.

**Conclusions:** Surgical resection of solitary lung metastases from CRC is associated with prolonged survival. High value of CEA, PET positivity, previous extrathoracic resected metastasis, and short (<12 months) DFI were found to be predictive of death or disease recurrence and might identify in this scenario patients at higher risk which could potential benefit of chemotherapy.

## Background

Colorectal Cancer (CRC) is one of the most frequent tumors as well as one of the major causes of death in developed Countries (1). Lung seeding occurs in ~10% of patients surgically treated for primary CRC with radical intent (2): lung is indeed the most common site of metastases after the liver.

Metastatic patients were managed with chemotherapy and/or best supportive cares, with unsatisfactory long-term prognosis (worse than 5% at 5 years); the development of new drugs (irinotecan and biological agents targeting EGFR or VEGFR) remarkably improved overall on disease-free survival in stage IV CRC (3).

The potential benefit of lung metastasectomy in CRC emerged firstly in 1997 from International Registry of Lung Metastasectomy consisting of 5,206 patients from Europe and North America, providing evidence of better survival in radically resected CRC Lung Metastasis (CRCLM) (4). Since this report, a growing number of studies accumulated supporting the potential therapeutic role of CRCLM resection, making it a widely accepted option in the multimodal treatment of metastatic CRC. Despite such evidence, most studies suffer from several limitations, such fairly small and heterogeneous cohorts and long period of inclusion (thus resulting in a subsequent biases due to evolution of clinical practice, diagnostic assessments, and chemotherapic drugs).

Currently, indications to CRC lung metastasectomy are given by NCCN guidelines which point out the following criteria: (1) radically (R0) resectable lung disease, (2) adequate lung function to tolerate lung resections, (3) control of primary tumor, and (4) absence of extrathoracic disease—except of liver metastases. However, it is still unclear which are the best candidates which could benefit from CRCLM resection and, among them who are those with higher risk of recurrence. Several studies have been reported as the number of metastases could influence the choice in performing or not the adjuvant treatment, with patients with multiple lung metastases more likely addressed to it.

To better clarify the potential role of adjuvant chemotherapy, even in patients with single lung metastases (SLM) from CRC, we analyzed the risk of recurrence (and long-term survivals) in a large cohort of patients surgically operated for SLM from CRC in 4 high volume centers. The results are reported herein.

## Patients and Methods

All consecutive patients who received operations for SLM from CRC between 1/2000 and 12/2016 in 4 centers were included. The centers were Azienda USL—IRCCS di Reggio Emilia, Hospital S. Camillo Forlanini (Rome), University of Pisa (Pisa), and Helsinki University Hospital, Heart and Lung Center (Helsinki, Finland). Demographic, clinical, surgical, and pathological data were retrospectively collected from the surgical registry. Follow-up was obtained by outpatient visits or telephone contact and was completed by 12/2017.

Indications for lung metastasectomy were given according to NCCN criteria (see above). Pre-operative patients' assessment included clinical examination, blood tests, electrocardiogram, computed tomography (CT) scan of the chest, and abdomen and whole-body positron emission tomography (PET) scan. Pulmonary function tests with diffusion capacity and arterial blood gas analysis were also performed to determine the feasibility of surgery and the extension of pulmonary resection.

The type of surgical approach was chosen according to size and location of pulmonary nodule: in most cases a muscle-sparing lateral thoracotomy was performed in order to palpate the lung. Peripheral lesions were treated by wedge resections, deep nodules were resected by laser-assisted enucleoresection or anatomical segmentectomy while larger hilar lesions required lobectomy. Nodal sampling was performed in case of enlarged lymph nodes or suspect FDG uptake at PET-Ct scan. Resection was defined as complete when microscopically negative (R0) margin was achieved. A threshold of 2.5 for SuvMax (as well as for NSCLC) was used to consider lung metastases as positive at PET-CT scan. Adjuvant CHT after resection of lung metastasis consisted in most of cases of oxaliplatin-based doublets [mFOLFOX: Folinic acid (leucovorin), 5-Fluorouracil, Oxaliplatin or CAPEOX: capecitabine and Oxaliplatin] or topoisomerase inhibitor (mFOLFIRI: Folinic acid, 5-Fluorouracile, Irinotecan) administered for approximately 6 months. Indication to adjuvant CHT was given according to stage of primary CRC, previous other extrathoracic metastases and previous CHT for primary CRC.

Before undertaking our data analysis, we obtained the IRCCS-Arcispedale Santa Maria Nuova (Promoting Center) the Institutional Review Board approval for research use of retrospectively collected data (observational) stemming from standard clinical practice.

### Data Variables and Outcomes

Overall Survival (OS) was the primary outcome and was computed from the date of lung surgery to the date of death (from any cause): for censored cases, the date of last available follow-up was considered. Perioperative mortality (within 30 days from operation) was considered for survival analysis.

Disease-Free Survival (DFS) was calculated from time of primary colonic surgery to time of lung surgery. Demographic, clinical, and pathological data included: age, gender, tumor size, blood levels of Carcino-Embryonic Antigen (CEA), FDG-PET scan findings, previous extrapulmonary metastasectomy, lung lesion size, type of surgical approach, extension of lung resection, nodal dissection.

Categorical variables were tested by Chi-square test. Survival curves were computed using the Kaplan-Meier method and compared by log-rank test at univariate analysis. Possible predictors of survival were investigated in multivariate analysis using the Cox proportional hazards model. Hazard ratios (HR) were provided with the corresponding 95% confidence intervals (95% CI). Multivariate analysis of disease recurrence was performed including prognostic factors in binary logistic regression analysis. Multivariate models were built with covariates with significant or borderline significant *p*-value (<0.2) at univariate analysis.

SPSS release 23.0 (SPSS inc.—Chicago, Illinois) was used for statistical analysis. A *p* ≤ 0.05 was accepted for significance.

## Results

A total of 344 patients were included in the study. The main demographic, clinic, and pathological features are summarized in Table 1.

**Table 1:** Clinical, radiological, and histological characteristics of the population.

**Character**	**Value**
Population, *n*	344
**Gender** ***n*****, %**	
M	196 (57.0)
F	148 (43.0)
**Access**	
Open	208 (61.0)
Converted VATS	15 (4.4)
VATS/RATS	118 (34.6)
**Type of resection**	
Wedge Resection	268 (77.9)
Lobectomy	74 (21.5)
Pneumectomy	2 (0.6)
**Tumor size (cm)**	
<2 cm	168 (49.1)
>2 cm	174 (50.9)
***N*** **Dissection**	
Y	64 (32.7)
N	132 (67.3)
**Neoadjuvant**	
Y	25 (7.9)
N	291 (92.1)
**Adjuvant**	
Y	115 (64.2)
N	64 (35.8)
**Prior extrathoracic metastasis**	
Y	65 (18.9)
N	279 (8.1)
**CEA**	
<5 ng/ml	75 (43.6)
>5 ng/ml	97 (56.4)
**PET**	
Positive	47 (40.2)
Negative	70 (59.8)

All patients had previously undergone radical resection of primary CRC: in most of cases (94.8%) lung metastases were found during follow-up, with a median Disease-Free Interval (DFI) of 35 months. In the remaining cases lung metastases were discovered during primary CRC staging. Sixty-four patients (35.8%) had been operated for extrathoracic (mainly hepatic) metastases too.

PET-Ct scan was performed prior to lung resection in 117 patients according to internal staging protocols and was positive in 47 (0.2%). The preferred surgical approach was sub-lobar resection, performed through lateral muscle-sparing thoracotomy (61%), although VATS was employed in a considerable percentage of patients (34.6%) for peripheral lung lesions. Major postoperative complications developed in 25 patients (7.6%), no deaths occurred within 30 days.

Adjuvant chemotherapy (various drugs regimens) was administered in the majority of patients (64.2%), while neoadjuvant (i.e., prior of lung surgery) only in 7.9% (mainly in synchronous lung metastasis). Adjuvant CHT was delivered in a different proportion of patients according to primary CRC TNM/UICC stage (*p* < 0.001) and previously resected extrathoracic metastasis (38/45 vs. 77/134, *p* < 0.001), while hilar nodal involvement (3/3 of N+ vs. 77/105 of N0), higher CEA (36/75 vs. 74/97), and short DFI (20/25 vs. 95/154) where not statistically associated to adjuvant CHT administration.

### Outcomes

**Figure 1:**
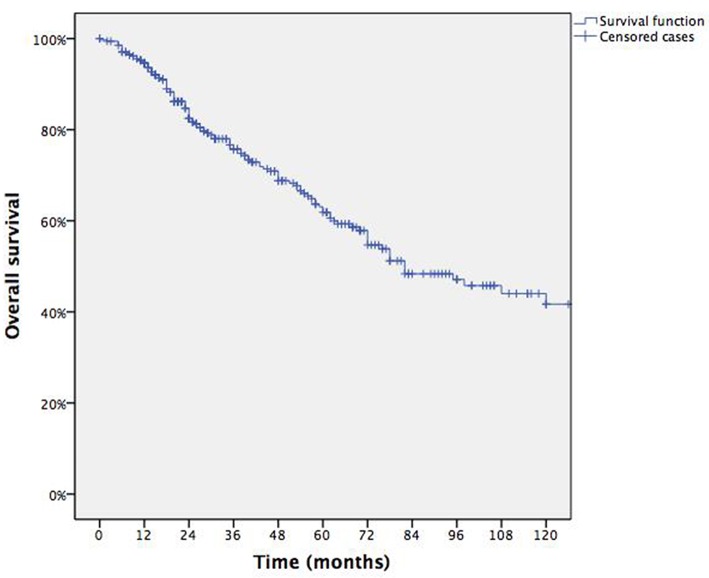
Overall survival.

**Figure 2:**
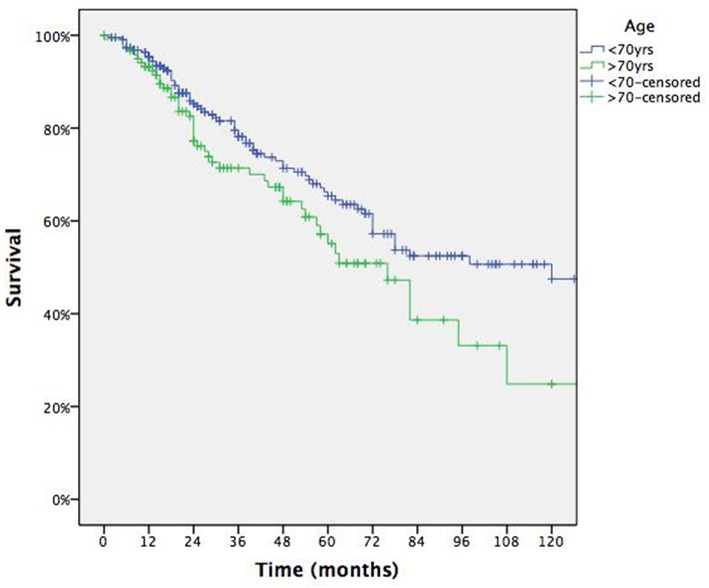
Survival according to age.

**Figure 3:**
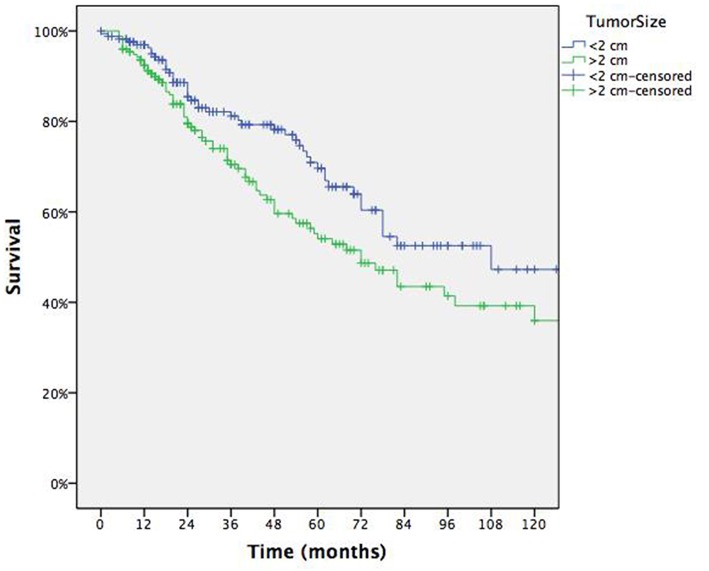
Survival according to tumor size.

Overall 5 yrs survival rate was 61.9% with median survival of 82 months (95% CI: range 61–103) for each patient (Figure 1). Table 2 summarizes survival data according to risk factors, showing Age > 70 yrs (Figure 2), Tumor size > 2 cm (Figure 3) and lung relapse after metastasectomy as significant predictors of death.

**Table 2:** Univariate analysis of survival.

**Factors**	**#pt**	**% 5 y**	**Median (Months)**	**Conf. range (95%)**	***p*-value**
OS	344	61.9	82	61–103	
**Gender**					
M	196	63.2	82	56–108	0.940
F	148	60.0	78	43–113	
**Age**					
<70 y	224	65.4	120	73–167	**0.046**
>70 y	120	55.1	76	59–93	
**Access**					
Open	208	59.7	78	57–99	0.116
Converted VATS	15	41.5	51	9–93	
VATS/RATS	118	66.7	–	–	
**Type of resection**					
Wedge Resection	268	61.3	78	58–98	0.150
Lobectomy	74	65.5	120	58–182	
Pneumectomy	2	0.0	26	–	
**Tumor size (cm)**					
<2 cm	168	69.6	108	77-139	**0.038**
>2 cm	174	54.1	72	53-91	
***N*** **Dissection**					
Y	64	65.0	95	65–125	0.881
N	132	68.0	98	67–130	
**Lung metastasis**					
Synchronous	18	52.8	78	40–116	0.457
Metachronous	326	62.5	82	60–104	
**Neoadjuvant CHT**					
Y	25	59.7	78	45–112	0.307
N	291	61.2	82	60–104	
**Adjuvant CHT**					
Y	115	63.5	78	56–100	0.773
N	64	65.0	95	64–126	
**Prior extrathoracic metastasis**					
Y	65	61.2	72	42–102	0.597
N	279	61.3	82	51–113	
**CEA**					
<5 ng/ml	75	73.0	95	65–125	0.120
>5 ng/ml	97	57.1	78	58–98	
**PET**					
Positive	47	59.4	–	–	0.238
Negative	70	72.6	–	–	
**DFI 12 months**					
<12	43	59.5	–	–	0.572
>12	301	62.3	82	61–103	
**DFI 24 months**					
<24	113	62.8	82	72–93	0.451
>24	231	61.1	82	47–117	
**DFI 36 months**					
<36	174	59.7	78	66–90	0.665
>36	170	63.2	98	56–140	
**Recurrence**					
Y	113	46.9	58	39–77	**<0.001**
N	227	70.0	108	–	
**Primary CRC stage**					
I	60	67.9	95	–	0.843
II	92	58.8	82	65–99	
III	146	59.6	72	32–112	
IV	36	59.3	72	23–121	
**Nodal status**					
N0	159	65.3	95	58–132	0.458
N+	13	51.3	–	–	
PET					
Performed	89	67.8	–	–	0.512
Not performed	83	61.5	82	38–126	

*Bold values refers to variables with p value under statistical significance threshold*.

At the end of the study period, 108 pts had lung relapse: synchronous (i.e., detected at time of diagnosis and staging of primary CRC) pulmonary metastases (*p* = 0.039), previous extrathoracic (mainly hepatic) metastases (*p* = 0.017), PET positivity (*p* = 0.006), and short DFI (<12 months) between CRC and first detection of LM (*p* = 0.048) were predictive of recurrence (Table 3).

**Table 3:** Univariate analysis of risk factors for lung recurrence.

**Factor**	**Recurrence**		***p***
	**Yes (%)**	**No (%)**	
**Age**			
<70 y	75 (33.8)	147 (66.2)	0.768
>70 y	38 (32.2)	80 (67.8)	
**Access**			
Open	72 (34.8)	135 (65.2)	0.451
Converted VATS	6 (40)	9 (60)	
VATS/RATS	33 (28.7)	82 (71.3)	
**Type of resection**			
Wedge Resection	86 (32.6)	178 (67.4)	0.497
Lobectomy	28 (37.3)	47 (62.7)	
Pneumonectomy	0 (0)	2 (100)	
**Tumor size (cm)**			
<2	48 (28.9)	118 (71.1)	0.105
>2	64 (37.2)	108 (62.8)	
***N*** **Dissection**			
Y	23 (36.5)	40 (63.5)	0.425
N	41 (31.8)	88 (68.2)	
**Pulmonary metastasis**			
Synchronous	10 (55.6)	8 (44.4)	**0.039**
Metachronous	103 (32.0)	219 (68.0)	
**Neoadjuvant CHT**			
Y	8 (32)	17 (68)	0.748
N	101 (35.2)	186 (64.8)	
**Adjuvant CHT**			
Y	38 (34.2)	73 (65.8)	0.522
N	25 (39.1)	39 (60.9)	
**Prior extrathoracic metastasis**			
Y	29 (46.0)	34 (54.0)	**0.017**
N	84 (30.3)	193 (69.7)	
**CEA**			
<5 ng/ml	22 (30.1)	51 (69.9)	0.137
>5 ng/ml	40 (41.2)	57 (58.8)	
**PET**			
Positive	19 (41.3)	27 (58.7)	**0.006**
Negative	12 (17.9)	55 (82.1)	
**DFI 12**			
<12	20 (46.5)	23 (53.5)	**0.048**
>12	93 (31.4)	203 (68.6)	
**DFI 24**			
<24	40 (35.4)	73 (64.6)	0.550
>24	73 (32.2)	154 (67.8)	
**DFI 36**			
<36	55 (31.6)	119 (68.4)	0.515
>36	58 (34.9)	108 (65.1)	

*Bold values refers to variables with p value under statistical significance threshold*.

**Figure 4:**
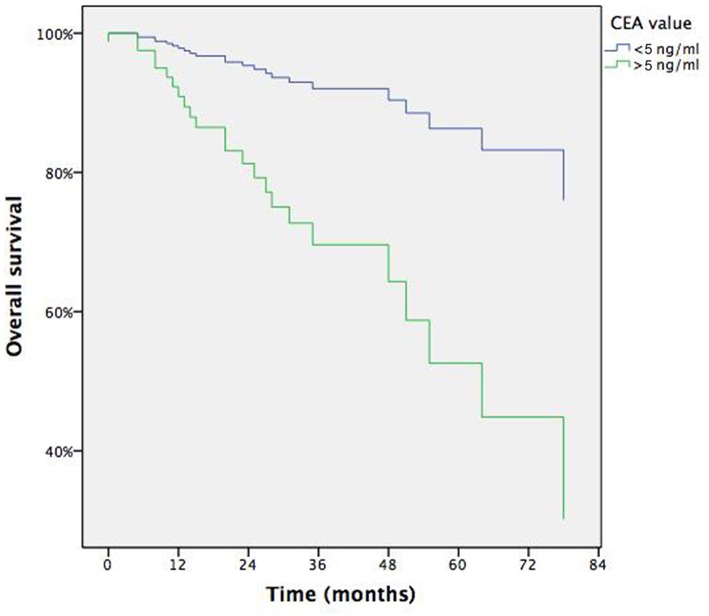
Survival according to CEA at means of covariates.

Multivariable analysis adjusted for covariates indicated that only elevated serum CEA level (>4 ng/ml) was predictive of worst survival with risk of death of 4.364 (95% CI: 1.356–14.050— *p* = 0.014), while previous extrathoracic metastasectomy (R.R.: 3.028, 95% CI: 1.091–8.406— *p* = 0.033), PET positivity (R.R.: 2.702, 95% CI: 1.041–7.013— *p* = 0.041) and DFI > 12 months (R.R.: 0.137, 95% CI: 0.054–0.346—*p* < 0.001) between CRC and first detection of LM confirmed to be predictive factor of recurrence. Table 4 summarizes results of Cox-regression and binary logistic regression, Figure 4 displays survival curves according to CEA levels at mean of covariates.

**Table 4:** Multivariate analysis of survival and recurrence.

	**Hazard ratio**	**CI (95%)**	***p*-value**
**Survival**			
Age > 70 y	1.300	0.502–3.366	0.589
Tumor size > 2 cm	1.405	0.553–3.569	0.475
Access: Converted VATS vs. thoracotomy	2.666	0.748–9.499	0.130
Access: VATS/RATS vs. thoracotomy	1.052	0.256–4.317	0.944
Lobectomy vs. sublobar resection	0.746	0.185–3.014	0.681
CEA > 5 ng/ml	4.364	1.356–14.050	0.014
PET positivity	1.673	0.479–5.846	0.420
**Recurrence**			
Tumor size > 2 cm	1.490	0.575–3.863	0.412
Synchronous pulmonary metastasis	0.817	0.186–3.584	0.789
Prior extrapulmonary metastasectomy	3.028	1.091–8.406	0.033
CEA > 5 ng/ml	0.900	0.366–2.215	0.819
PET positivity	2.702	1.041–7.013	0.041
DFI > 12 months	0.137	0.054–0.346	<0.001

## Discussion

After curative resection of CCR, more than 50% of the patients had recurrence of the disease, mostly in the liver and the lung (5). Over the last decade, lung metastasectomy has a valid option of curative intent in the context of a multimodal treatment if a series of criteria are met. These prerequisites were first described by Thomford in 1965 and are widely applied today, with minor modification (6): (1) technical feasibility of metastases resection; (2) fitness for surgical intervention scheduled; (3) control of the primary tumor; and (4) no evidence of extra-thoracic disease. However, controversies remain on indication, timing and modality of surgery worldwide, besides indication to complementary systemic treatments (chemotherapy).

More robust evidences are provided by literature about the importance of the number of metastatic lung lesions. Several authors have reported the number of metastasis as a predictive factor of recurrence and long-term survival. Pfannschmidt et al. in a retrospective cohort study stated that a low number of metastases (<4) could qualify patients for lung metastasectomy (7). Cho et al., in a recent paper focused on this topic, concluded that overall survival and recurrence after pulmonary metastasectomy for CRC is dependent on the number of metastases and surgical treatment can be offered to patients with three or fewer pulmonary metastases (8). Most authors however found that the best discriminant for long survival, after lung surgery for CRCLM, is the presence of single vs. multiple metastases (9–12).

These results have been corroborated by a recent meta-analysis conducted by Gonzalez for a total amount of 2,925 patients in which the presence of more than one lung lesion was associated to an higher risk of death (HR = 2.04, 95% CI: 1.72–2.41) in 20 of 25 studies considered, giving level of evidence 1a to surgical resection as therapy of choice for treatment of single metastatic pulmonary nodule.

In the present cohort, overall survival at 5 years after resection of a unique CRCLM reached an encouraging result of 61.9%. At univariate survival analysis, higher age and tumor size demonstrated a negative impact on survival (Table 2). Age is reported in two studies as significant prognostic factor, either as a discrete variable (> or <70 yrs) (8) than as a continuous variable (5). Tumor size has been rarely reported as prognostic factor since many of the studies include multiple metastases, however in a large cohort from Japan, T size > 2 cm was a strong predictor of worst survival (HR = 1.577, 95% CI: 1.262–1.971, *P* < 0.0001) (13). In the multivariable analysis both these factors lost significance and the unique predictor resulted the CEA level before lung surgery (Table 4). CEA may be considered as an indicator of burden of disease, either macroscopical than microscopical-subclinical, although its level depends on other factors such as differentiation. Anyway many studies report pre-lung resection CEA among prognostic factors (5, 7, 11, 13–15) but authors advocate the use of CEA as a follow-up tool rather than a selection criterium for metastasectomy if radical (R0) resection is deemed feasible.

The real gap of knowledge in the current literature regards the risk of recurrence after resection for single CRCLM, this limiting the selection of the best candidates for adjuvant treatment. The predictive role of recurrence of pre-op PET/CT in single CRCLM has been only rarely reported because of it was not routinely executed in the past years. In the present series evaluating the 117 patients who underwent whole body PET-CT scan prior to lung resection, the increased FDG uptake (SUVMax>2.5) seems to increase risk of recurrence after lung metastasectomy.

Therefore, the execution of PET scan could potentially have more than one purpose: (1) to achieve metabolic information about the pulmonary nodule during the diagnostic work-up examination, (2) to exclude the presence of other sites of metastases and of mediastinal nodal disease, (3) to identify those patients who could benefit from adjuvant treatment after surgery.

At the same time, it's interesting to note that in this series, the administration of CHT does not improve the long-term survival, neither in neoadjuvant nor adjuvant setting. The data in literature are almost scarce and discordant in this setting; the neoadjuvant CHT is reported to improve survival in two studies (8, 16) only, while no significant impact is reported for adjuvant CHT at today. Patients who develop a lung metastasis or who progress under CHT treatment were found to have a worse chance of survival (10, 17). In the present cohort, survival curves according to administration or not of neoadjuvant/adjuvant CHT were comparable, with a slight advantage for patients who did not receive any treatment; this paradoxical effect is commonly seen in retrospective series and reflects the fact that CHT is proposed in patients judged at higher risk. In fact, as reported in the results, adjuvant CHT was delivered to patients with higher stage primary CRC (III/IV) and previous extrathoracic (hepatic, in most cases) metastasectomy. However, these data provide evidence that there is variability in indications to CHT in this setting, in part due to multicentric nature of the study.

We also found as the presence of a previous metastases, a short DFI (<12 months) or the occurrence of lung metastasis synchronously to the primary CRC are predictive factors for further recurrence after lung resection for single CRCLM. It's seems to be logical that these are factors directly correlated with the biological behavior of the disease. In fact, from a logical point of view, a scenario where a pulmonary localization occurred in the same time or few months later, the diagnosis of primary CRC (and maybe with a liver metastases resected at the same time of colon resection) is the expression of a different biological behavior when compared with another scenario where a single pulmonary metastase appeared several years after treatment of CRC. Physicians should take into account this information when planning a multimodal strategy of care in these patients.

A final consideration about surgical approach could be done: our results support that a VATS approach had comparable results to open approach, as stated from a recent paper. Only converted VATS (due to failure in localizing lung metastasis or incomplete resection with positive margins) seemed to increase the risk of local recurrence. These data support the fact that a VATS approach is feasible for peripheral lung lesions: in this case a preoperative (very close to the date of operation) HRCT may help to better selection of patients resectable by VATS and avoid unnecessary lung palpation for localizing other misunderstood nodules (18).

## Conclusions

Despite some limitations and weak points of this study, which suggest that care is taken when interpreting results, as a higher CEA level; a history of previous extrathoracic treated metastasis; and a short DFI and PET positivity of CRCLM identify a subgroup of patients who are at high risk of death or re-recurrence. These prognostic factors may be useful in clinical practice for either defining a policy for administration of chemotherapy in a neoadjuvant or adjuvant setting, either for better selecting of patient candidates to extended resections (lobectomy or pneumonectomy) for single lung metastases. Further studies are needed to support the present data.

## Data Availability

The datasets generated for this study are available on request to the corresponding author.

## Author Contributions

CR conceived the design of the study, performed statistical analysis, and drafted the manuscript. FL made the design of the study and drafted the manuscript. FD, FC, SR, and JK contributed to database building. MP, FM, JR, and GC revised the manuscript. TD revised the database and checked it before analysis. All authors read and approved the final manuscript.

### Conflict of Interest Statement

The authors declare that the research was conducted in the absence of any commercial or financial relationships that could be construed as a potential conflict of interest.
